# Supplementary figures and images for: Antimicrobial Resistance and Whole-Genome Characterisation of High-Level Ciprofloxacin-Resistant Salmonella Enterica Serovar Kentucky ST 198 Strains Isolated from Human in Poland
Source: Int J Mol Sci. 2021 Aug 29;22(17):9381. doi: 10.3390/ijms22179381 (PMC8431004; doi:10.3390/ijms22179381)

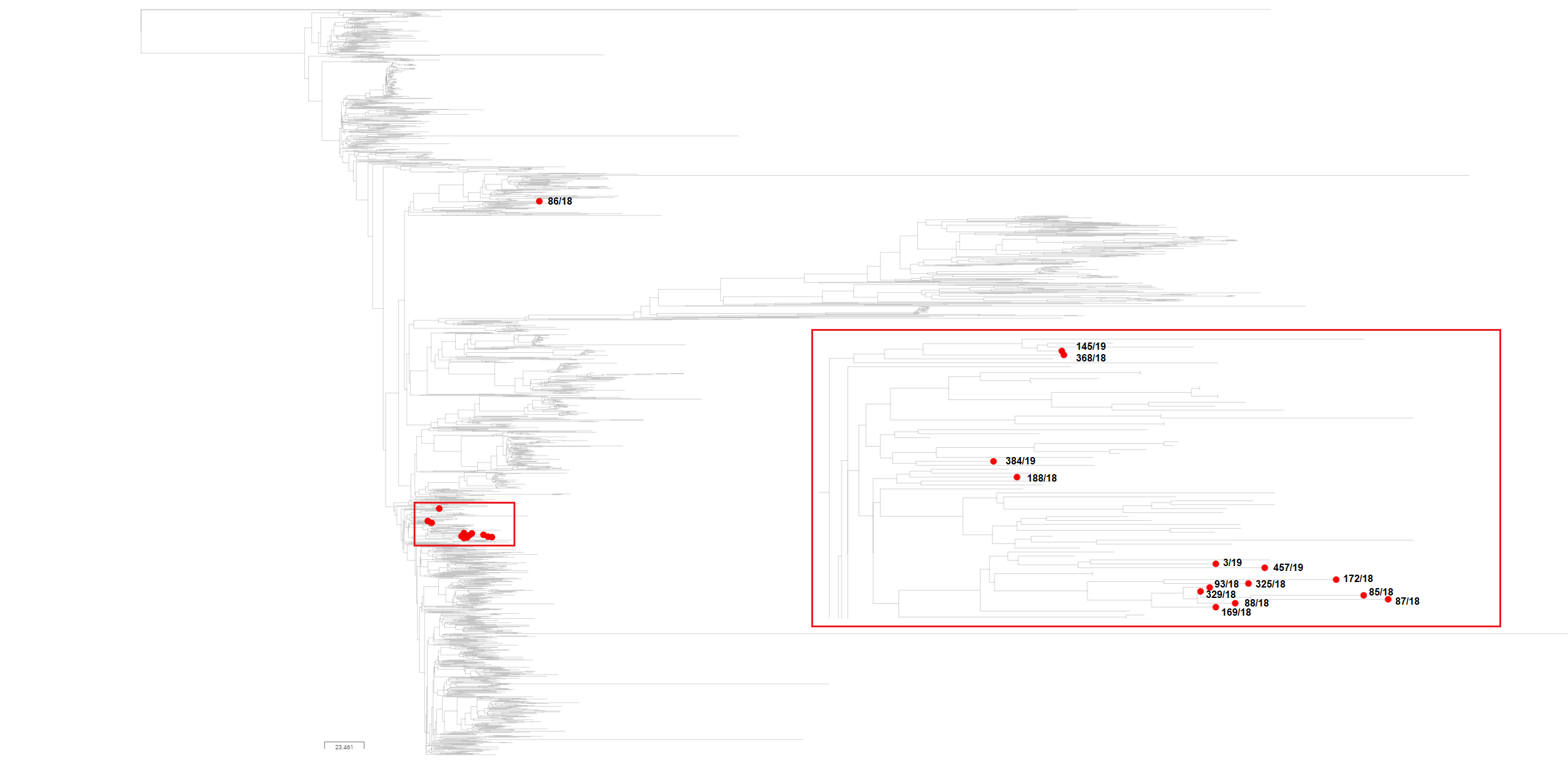

Supplement: Supplementary file 1 [file ijms-22-09381-s001.zip › Supplementary Files - S. Kentucky global wgMLST/S.Kentucky ST198 wgMLST - graphical phylogenetic tree - global analysis.tif]

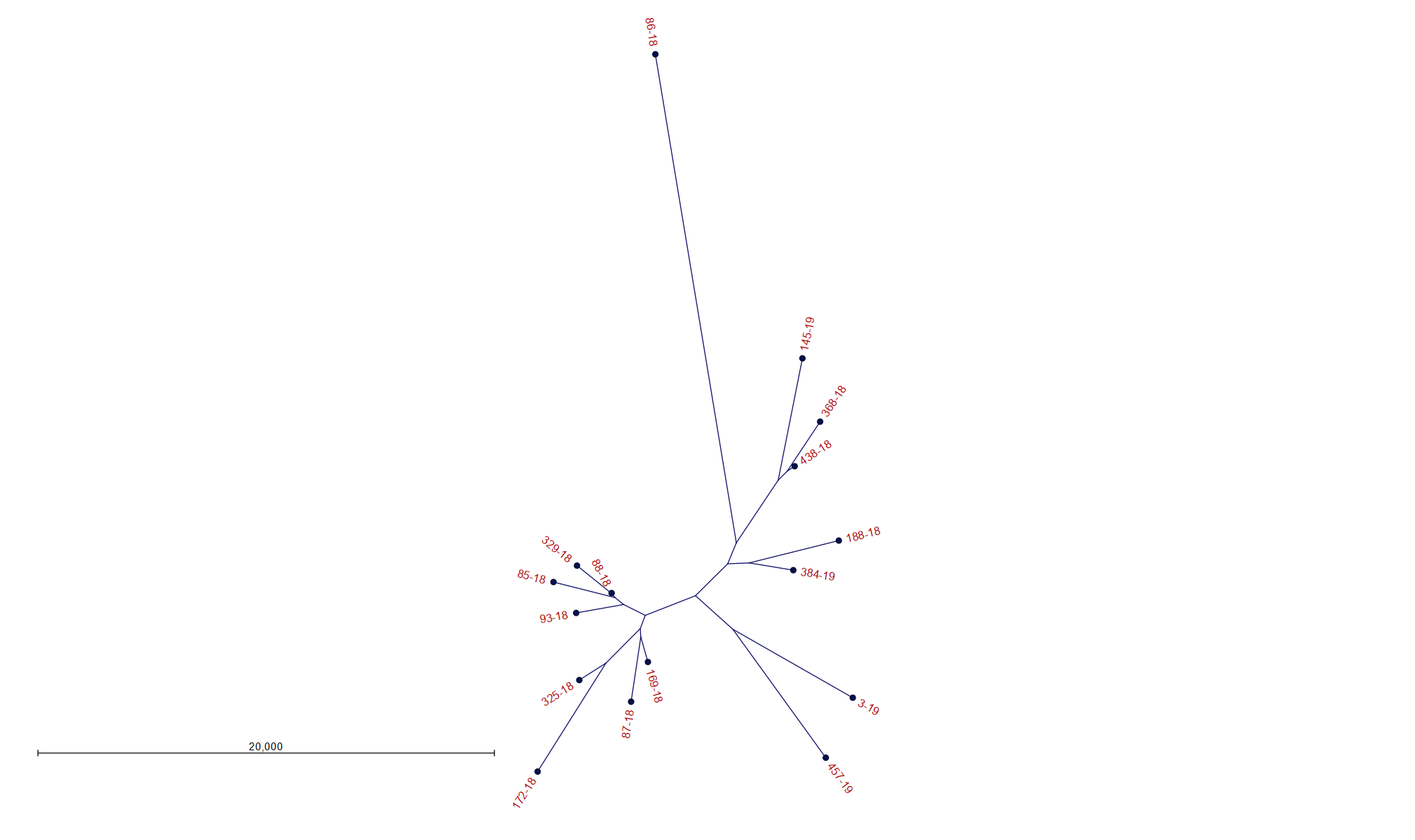

Supplement: Supplementary file 1 [file ijms-22-09381-s001.zip › Supplementary Files - S. Kentucky global wgMLST/S.Kentucky ST198 wgMLST - graphical phylogenetic tree - local analysis.tif]
